# Criteria required for an acceptable point-of-care test for UTI detection: Obtaining consensus using the Delphi technique

**DOI:** 10.1371/journal.pone.0198595

**Published:** 2018-06-07

**Authors:** Nichola-Jane M. Weir, Sally H. Pattison, Paddy Kearney, Bob Stafford, Gerard J. Gormley, Martin A. Crockard, Deirdre F. Gilpin, Michael M. Tunney, Carmel M. Hughes

**Affiliations:** 1 School of Pharmacy, Queen’s University Belfast, Belfast, Northern Ireland; 2 Centre for Experimental Medicine, Queen’s University Belfast, Belfast, Northern Ireland; 3 Northern Health and Social Care Trust, Bretten Hall, Antrim, Northern Ireland; 4 Orchard Carehomes, The Hamlet, Hornbeam Park, Harrogate England; 5 Department of General Practice, Queen’s University Belfast, Dunluce Health Centre, Belfast, Ireland; 6 Randox Laboratories Ltd, Crumlin, Northern Ireland; GGD Amsterdam, NETHERLANDS

## Abstract

**Background:**

Urinary Tract Infections (UTIs) are common bacterial infections, second only to respiratory tract infections and particularly prevalent within primary care. Conventional detection of UTIs is culture, however, return of results can take between 24 and 72 hours. The introduction of a point of care (POC) test would allow for more timely identification of UTIs, facilitating improved, targeted treatment. This study aimed to obtain consensus on the criteria required for a POC UTI test, to meet patient need within primary care.

**Methods:**

Criteria for consideration were compiled by the research team. These criteria were validated through a two-round Delphi process, utilising an expert panel of healthcare professionals from across Europe and United States of America. Using web-based questionnaires, panellists recorded their level of agreement with each criterion based on a 5-point Likert Scale, with space for comments. Using median response, interquartile range and comments provided, criteria were accepted/rejected/revised depending on pre-agreed cut-off scores.

**Results:**

The first round questionnaire presented thirty-three criteria to the panel, of which 22 were accepted. Consensus was not achieved for the remaining 11 criteria. Following response review, one criterion was removed, while after revision, the remaining 10 criteria entered the second round. Of these, four were subsequently accepted, resulting in 26 criteria considered appropriate for a POC test to detect urinary infections.

**Conclusion:**

This study generated an approved set of criteria for a POC test to detect urinary infections. Criteria acceptance and comments provided by the healthcare professionals also supports the development of a multiplex point of care UTI test.

## Introduction

Collectively, Urinary Tract Infections (UTIs) represent the second most common bacterial infections that occur within primary care, preceded only by respiratory infections.[1] Management and treatment of UTIs is an enormous burden to healthcare systems accounting for 1–3% of all general practitioner (GP) consultations within primary care[2] and approximately 30–50% of all antimicrobial prescriptions.[1] The predominant urinary pathogen is *Escherichia coli*, responsible for 60–80% of all UTIs.[3,4] Culture remains the gold standard for the identification of bacterial species.[5] However, it is slow, requiring 24–72 hours to report both the microorganism and provide an antibiotic resistance profile.[6] In primary healthcare, prescriptions are often prescribed based on presentation alone, without use of a diagnostic test, or are based on dipstick tests, which lack accuracy and give no indication as to the infecting agent.

In primary healthcare settings, in particular, there is an unmet clinical need to provide a more rapid and accurate infection detection test, thus there is an opportunity for the introduction of a point-of-care (POC) test. Point-of-care testing is defined as ‘laboratory diagnostic testing, performed at or near the site where clinical care is delivered’.[7,8] An ‘ideal’ POC test should be based on the needs of the user and provide the healthcare professional with the required information to enable appropriate treatment to be given to patients.[9] The test has to be sufficiently accurate to meet the purpose for which it has been designed.[9]

The ASSURED guidelines have been specifically developed for new POC tests for Sexually Transmitted Infections (STIs) and are as follows: Affordable—to those at risk of infection; Sensitive—reduce false negative results; Specific—reduce false positive results; User-friendly—minimal steps and use of non-invasive specimens; Rapid and Robust—short turnaround time and room temperature storage conditions; Equipment-free–minimal equipment required to ensure ease of use; Delivered–accessible to end-users.[10] As no similar criteria are available for a POC UTI test, this study aimed to develop and obtain consensus on the criteria required for such a test for these common infections.

## Methods

### Study design

A Delphi consensus technique was used in this study. This technique is a long established method which allows informed decision-making, in areas of research where there is little information, through obtaining consensus from expert opinions.[11,12] Ethical approval for this study was obtained from the School of Pharmacy, Queen’s University Belfast in May 2016.

### Compilation of initial criteria

The Steering Group, which consisted of nine members (authors on this paper), developed and reviewed a proposed set of criteria in the form of statements, associated with an acceptable point-of-care test. Sources for such criteria included peer-reviewed scientific literature, the ASSURED guidelines, clinical and laboratory guidelines, manufacturers’ literature, regulatory authorities and knowledge and experience from within the Steering Group. Previous studies investigating POC testing for various infections including STIs and Human Immunodeficiency Virus (HIV) informed the development of the criteria. In these studies, the areas which were highlighted as important included an easy-to-use test [13,14], high accuracy [15] and achieving a result a shorter turnaround time [13]. Each of the ASSURED guidelines were added to the initial criteria [10]. Clinical and laboratory guidelines were used to identify which micro-organisms to target with the POC test and the total load [Colony Forming Unit (CFU/ml)] at which these micro-organisms are considered clinically relevant in a urine sample. The developed criteria were grouped into five sections: intended use of the point-of-care test; the detection and identification of potential urinary pathogens; features and performance of the point-of-care device; operation of the point-of-care test by user and costs associated with the point-of-care test.

### Selection of the Delphi panel

Each Steering Group member was requested to identify at least five experts, with recognised expertise in their clinical area, as potential participants in the Delphi panel. Forty-two potential participants were identified from a range of healthcare professionals, ensuring representation from across several specialities including general practitioners (GPs), microbiologists, medical microbiologists, pharmacists, nurses and nursing home directors. The panel, representing geographical regions from across Europe and the United States of America were asked to respond to the Delphi questionnaire via email. Of 42 experts invited, 17 agreed to participate, which is sufficient for Delphi studies of this type.[16]^,^[17] These participants were representative of both the specialities and location of those invited, details of which are presented below in Table 1.

**Table 1:** Professional background and geographical location of the participating Delphi panel members.

Profession	Number of panel members
Microbiologists	2
Physician microbiologists	2
GPs	6
Pharmacists	2
Nurses	5
**Geographical location**	
United Kingdom (UK)	9
Republic of Ireland	5
Other parts of Europe	2
USA	1
**Total**	**17**

Reasons for non-participation were not sought. Written consent was received from those who had agreed to take part before the process commenced.

### Data collection and analyses

The Delphi process involved two rounds of web-based questionnaires (S1 and S2 Files). The questionnaire was piloted by pharmacist PhD students (n = 2), a microbiologist PhD student (n = 1) and a physician PhD student (n = 1) for usability and readability. Questions were revised according to their initial feedback. The first and second rounds of questionnaire distribution took place between May—August 2016 and between September—November 2016, respectively. For each round, panel members received an email with a link to an online survey tool (SurveyGizmo). Reminders were sent to panel members via e-mail to encourage completion of the survey. Members were asked to evaluate each statement using a five-point Likert scale[18] where 1 was “strongly disagree” and 5 was “strongly agree” and were encouraged to provide additional comments. Level of agreement for each statement was assessed using the median response and interquartile range (lower and upper quartile), and the following process was adhered to: when the upper quartile was less than 3, the statement was rejected as this indicated that consensus had not been reached; when the lower quartile was greater than 3, the statement was accepted as consensus had been reached. When the interquartile range included 3, this indicated a lack of agreement on the statement, and further consideration was necessary.[16] For these latter criteria, a review was carried out by the Steering Group and these were either revised and included in the second round or rejected based on comments provided by the experts or through the expertise within the Steering Group. The revised questionnaire was distributed to panel members who received their own individual and group responses. As before, the median and interquartile range was calculated and reviewed, along with comments provided by panel members. If consensus was not reached by the second round, the criterion was rejected.

## Results

**Fig 1:**
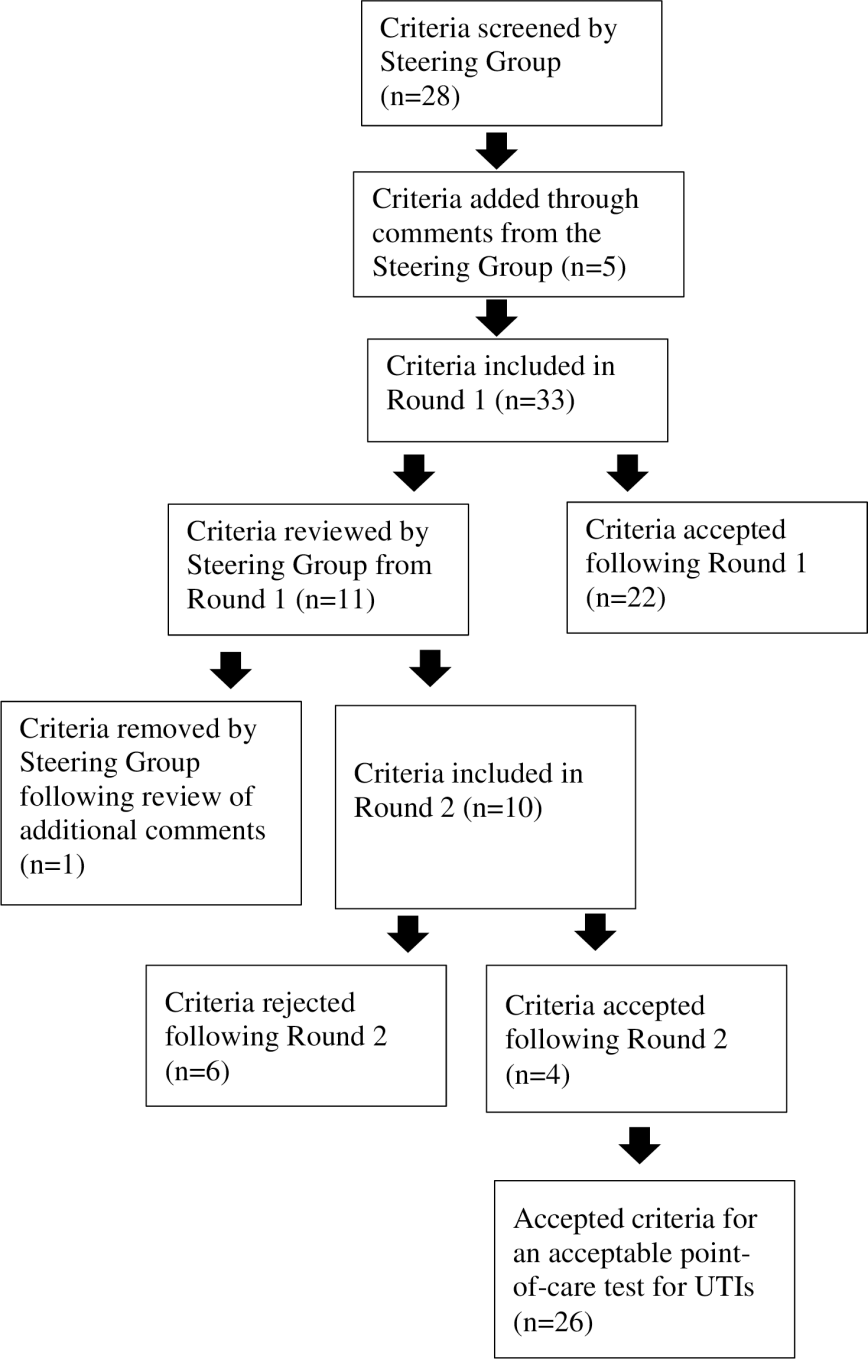
Flow diagram of the Delphi process which developed the criteria required for an acceptable point-of-care test for the detection of urinary pathogens.

A summary of the Delphi process is shown in Fig 1. Twenty eight criteria were developed using a range of sources (see above) and reviewed. Consultation within the steering group derived an additional five, thus thirty-three criteria were presented in the first round of the Delphi process. All 17 panel members who agreed to participate in this Delphi exercise completed the first round of the questionnaire. Group consensus (by overall agreement) was achieved for 22 criteria, which were accepted as criteria required for a POC test for UTIs. Consensus was not achieved for 11 criteria; these were reviewed and discussed by the Steering Group. This resulted in the removal of one criterion as it had been adequately covered by other criteria. Raw data from the Delphi questionnaire (Round 1) have been provided in S1 Table, and comments from the Round 1 survey have been provided in S3 File.

The second round of this exercise was completed by 15 members. Due to the anonymity of the exercise, we were unable to obtain the reasons for non-participation by two respondents in the second round. From Round 2 results, three criteria were accepted as part of the criteria required for a POC test for UTIs. Based on comments provided by the panel following Round 2, one criterion was re-worded.

Within this round, the remaining six criteria were rejected as no consensus had been reached. The main reason for rejection was based on uncertainty linked to POC test costs. The criteria which were rejected were: (1) The point-of-care test will require patients’ consent (where possible and practicable) for their urine specimen as occurs with all diagnostic tests; (2) Relevant healthcare professionals can be notified of results from the point-of-care test automatically via email as an optional feature; (3) Would you be willing to pay £30 (€38) per sample to detect and identify the most common urinary pathogens within 4 hours (cost includes the price of the point-of-care instrument)? (4) If the initial device cost less than £10,000 (€12,403) would you be interested in buying the device? (5) Would you be willing to pay an extra £5 (€6.30) per sample to achieve the test result in <2hours? (6) Would you be willing to pay an extra £2.50 (€3.10) per sample to detect genetic indicators of resistance to trimethoprim which may better inform appropriate antibiotic treatment?

Exemplar comments received from the panellists are shown in Table 2. Raw data from the Delphi questionnaire (Round 2) have been provided in S2 Table, and comments from the Round 2 survey have been provided in S4 File.

**Table 2:** Exemplar comments received from the Delphi panel.

Example of statement from Round 1	The point-of-care test can be used within secondary care for detection of urinary pathogens.	Staff operation of the point-of-care test will include: a. Collection of urine sample from the patient.
Comments from Round 1	• ‘Not so critical with laboratory on site and new MALDI-TOF technology for rapid identification. Difficulty remains with older patients who have chronic bacteriuria. What does POC test add to dipstick?’ • ‘Most patients with UTI are treated in primary care, and the need of POC here is highest. Secondary care has a number of other tests available.’	• ‘not sure what you mean by "collection". the patient would usually "collect" the sample and the staff would "receive and verify" the sample’ • ‘Patient or their career should ideally collect the sample’
Revised statement for Round 2 (**revisions are shown in bold**)	The point-of-care test can be used within secondary care **as a “one-step” test** for the detection and **identification** of urinary pathogens **when a faster result is required.** Explanation: **In comparison to a) Matrix-assisted laser desorption/ionization time-of-flight (MALDI-TOF) which identifies pathogens once detected and b) the dipstick which only detects the presence of a pathogen, this point-of-care test will detect and identify the urinary pathogens in a one-step process.**	Staff operation of the point-of-care test will include: -**Receipt and verification** of the urine sample from the patient
Comments from Round 2	• ‘This would be an ideal situation, not withstanding continued interpretation of the significance of any organism detected in urine’	• ‘Avoid error’ • ‘Ideally a point of care test will be carried out by a patient facing member of clinical staff such as a nurse or doctor.’
Conclusion	Accepted following the revision made for Round 2	Accepted following the revision made for Round 2

The final set of criteria required for an acceptable POC UTI test consisted of 26 items in the following categories: intended use of the point-of-care test (n = 4), detection and identification of potential urinary pathogens (n = 8), features and performance of the point-of-care device (n = 10) and the operation of the point-of-care test by user (n = 4). This set of accepted criteria are presented in Tables 3 and 4.

**Table 3:** Accepted criteria from Sections 1 and 2 for an acceptable point-of-care test for the detection of urinary pathogens.

STATEMENT NUMBER	STATEMENT
**Section 1: Intended use of the point-of-care test.**	
1	**The point-of-care test can be used for any patient suspected of having a UTI (i.e. presenting with symptoms, such as confusion, agitation, concentrated urine, dehydration) regardless of age/demographic.**
2	**The point-of-care test can be used within GP surgeries.** **Explanation:** Urine samples are often received at GP surgeries. Therefore, the point-of-care test will be sufficiently easy to use with minimal training requirements.
3	**The point-of-care test can be used within care home environments.** **Explanation:** UTIs are common within care home environments, this leads to a significant number of urine samples. The point-of-care test will be sufficiently easy to use with minimal training requirements.
4	**The point-of-care test can be used within secondary care as a “one-step” test for the detection and identification of urinary pathogens when a faster result is required.** **Explanation:** In comparison to a) Matrix-assisted laser desorption/ionization time-of-flight (MALDI-TOF) which identifies pathogens once detected and b) the dipstick which only detects the presence of a pathogen, this point-of-care test will detect and identify the urinary pathogens in a one-step process.
**Section 2: The detection and identification of potential urinary pathogens.**	
5	**Detection of the most common urinary pathogens will be achieved by the point-of-care test.** **Explanation:** Urinary pathogens for detection by the point-of-care test include: *Escherichia coli*, *Klebsiella pneumoniae*, *Klebsiella oxytoca*, *Enterococcus faecalis*, *Enterococcus faecium*, *Proteus mirabilis*, *Proteus vulgaris*, *Proteus penneri*, *Providencia stuartii*, *Providencia rettgeri*, *Morganella morganii*, *Staphylococcus saprophyticus*, *Pseudomonas aeruginosa*, *Candida albicans*, *Staphylococcus aureus*, *Enterobacter cloacae*, *Enterobacter aerogenes*, *Serratia marcescens*, *Citrobacter koseri*, *Citrobacter freundii*, *Acinetobacter baumannii*, Staphylococcus epidermidis and Streptococcus agalactiae*. Urinary pathogens for detection were chosen based on clinical and laboratory guidelines. *Streptococcus agalactiae was excluded from the criterion however it should be detected by the point-of-care test.
6	**Results obtained by the point-of-care test should have a high sensitivity.** **Explanation:** High probability of correctly detecting and identifying a urinary pathogen.
7	**Results obtained by the point-of-care test should have a high specificity.** **Explanation:** High probability of accurately identifying the absence of a urinary pathogen.
8	**Results obtained by the point-of-care test should have a high positive predictive value.** **Explanation:** Positive predictive value–probability that those samples which test positive with the point-of-care test truly have a urinary pathogen present.
9	**Results obtained by the point-of-care test should have a high negative predictive value.** **Explanation:** Negative predictive value–probability that those samples which do not test positive with the point-of-care test truly do not have a urinary pathogen present.
10	**The level of detection required by the point-of-care test for the urinary pathogens is from 10**^**2**^ **to greater than 10**^**5**^ **CFU/ml as determined by the published guidelines.** **Explanation:** Based on: 1. Clinical guidelines including Scottish Intercollegiate Guidelines (SIGN), European Association of Urology (EAU), and Infectious Disease Society of America (IDSA). 2. Laboratory guidelines including Cumulative techniques and procedures in clinical microbiology (Cumitech) and Public Health England.
11	**Identification of urinary pathogens should be to the genus or species level as appropriate.** **Explanation:** Identification of urinary pathogens will be at the same level or better than determined by conventional culture. Current culture methods do not typically identify non- *E*. *coli Enterobacteriaceae* to a more detailed level (e.g. genus or species) unless multi-resistance is present. However, the point-of-care test will improve this level of identification by reporting *Enterobacteriaceae* at the genus level, e.g. *Klebsiella spp*., *Citrobacter spp*., *Enterobacter spp*. or *Proteus spp*. or to species level e.g. *Morganella morganii*, *Acinetobacter baumannii*.
12	**Appropriate prescribing of antibiotics will be aided by the point-of-care test.** **Explanation:** Identification of urinary pathogens will be achieved in less than 4 hours. This would permit the prescription of an appropriate antibiotic by the GP, which could then be dispensed to the patient within 24 hours of the specimen being received. Currently if the urine sample is sent for culture, this typically is achieved within 3–4 days.

**Table 4:** Accepted criteria from Sections 3 and 4 for an acceptable point-of-care test for the detection of urinary pathogens.

STATEMENT NUMBER	STATEMENT
**Section 3: Features and performance of the point-of-care device**	
13	**Detection and identification of pathogens directly from the urine sample will be completed in a one-step process.** **Explanation:** Once a sample has been loaded onto the instrument, no further input is required from the staff member.
14	**The point-of-care test should offer quicker detection than conventional culture methods to better inform clinical decision making.** **Explanation:** Detection and identification of urinary pathogens will be completed within a more clinically relevant time frame.
15	**The point-of-care test should operate as a stand-alone instrument.** **Explanation:** Consumables for the point-of-care test will include: urine collection pot, disposable pipette, test reagent cartridge and the instrument.
16	**A small sample volume of urine will be required for the point-of-care test.** **Explanation:** The test will use approximately 1 ml of mid-stream urine. Mid-stream urine will be used to minimise contaminating flora.
17	**Only one sample will be analysed at a time, using the point-of-care test.**
18	**The space required for the point-of-care test instrument and operation should be minimal.** **Explanation:** Size of the instrument will be approximately 30cm x 60cm x 45cm (Width x Depth x Height).
19	**As part of the point-of-care test, unique barcode sample tracking will be provided.** **Explanation:** Samples will be identified using unique barcodes containing information such as patient name, date of birth, gender, patient identification number, name of general practitioner and date of sampling
20	**Results can be stored on the point-of-care test.** **Explanation:** Results will be stored on the point-of-care test as a backup and for audit purposes.
21	**Results can be printed directly from the point-of-care test.** **Explanation:** A hard copy of the results can be added to the patient’s clinical records.
22	**Results from the point-of-care test can be automatically added remotely to patient’s records as an optional feature.** **Explanation:** Optional because this will incur extra costs to cover integration of networking capabilities.
**Section 4: Operation of the point-of-care test by user**	
23	**Minimal staff training should be required to use the point-of-care test.** **Explanation:** Staff training will be provided to ensure accuracy and familiarity for the use of the instrument.
24a	**Staff operation of the point-of-care test will include the following steps:** **Receipt and verification of the urine sample from the patient**
24b	**Storage of the urine sample if required.** **Explanation:** Storage of the urine sample will only be required if there is a backlog of samples to be analysed.
24c	**Safe handling and loading of the urine sample onto the instrument.**
24d	**Input of sample information via the touch screen on the instrument.**
24e	**Safe disposal of the urine sample.**
25	**Maintenance and quality control will be required for the point-of-care instrument.** **Explanation:** Training will be provided for maintenance of the instrument and quality control procedures.
26	**The time required by staff (as detailed in statements 26a-e) to run the point-of-care test will be minimal.** **Explanation:** Staff time will be required for training, maintenance of equipment, quality control of the instrument and recording of the results.

## Discussion

Using a Delphi consensus method, we developed a list of criteria for an acceptable POC test for detection of urinary pathogens. The expert panel facilitated development of the criteria through their level of agreement, and revisions to statements were based on comments provided by panellists.

The 26 criteria encompassed several aspects of use: firstly, the intended application of the POC test. Within this section, consensus was achieved, indicating that the test could be used for any patient presenting with symptoms of a UTI in a GP surgery, care home environment or secondary care setting. Secondly, detection and identification of potential urinary pathogens; consensus was achieved regarding the most common urinary pathogens to be detected with high sensitivity and specificity. Thirdly, the features and performance of the POC device. The accepted criteria within this section highlighted how results will be generated from the test to allow quicker detection of urinary pathogens present. Finally, considerations concerning operation of the test by the user resulted in consensus in the number and complexity of steps required to operate the device by healthcare professionals. The methodology did not facilitate a ranking of criteria in terms of priority for users and future work could examine this. However, it may not be reasonable to expect one criterion to be ranked over and above all other criteria. In view of the complexity of a POC device and the range of features that may be required to produce a test with widespread acceptance, it may be necessary to rank criteria within groups of statements e.g. in statements relating to the intended use of the point-of-care test, which of the accepted criteria would be most important?

Previous qualitative research studies have contributed to the development of criteria required for point-of-care tests for STIs.[15] These have highlighted the main features and how the POC test should work for detection of STIs. The main themes were the pathogens to be identified, time required for the test and the need for high test accuracy.[15] Kaman et al[13] reported the requirements for POC tests for infectious diseases by medical personnel, manufacturers and the general public. Requirements were similar between manufacturers and medical personnel and included time to diagnosis, reliability and specificity.[13]

Howick et al[19] conducted a study across Australia, Belgium, the Netherlands, UK and USA and found an unmet need for POC testing amongst primary care doctors to help diagnose UTIs. Similarly 47% of GPs within the UK expressed a need for a more accurate test than the urine dipstick to aid the diagnosis of a UTI, which would subsequently help to reduce antibiotic prescription levels.[20] Within our study, consensus was achieved on a number of these issues by a range of healthcare professionals, suggesting similar interest for more accurate detection of UTIs.

The Horizon scan report for POC testing for UTIs suggested there is a need for the development of rapid pathogen identification.[21] Consensus achieved within the second section of the required criteria showed the importance of detecting the most common pathogens with high sensitivity and specificity, which has also been noted by the ASSURED guidelines for STI testing.[10]

Studies conducted by Kaman et al[13] and Nouvellet et al[22] suggested that the maximum number of processing steps should be between two and three and that the test should require minimal laboratory facilities and minimal staff training. Consensus was achieved within the Delphi panel indicating the importance of creating a device which allows identification of urinary pathogens direct from the urine sample, with minimal processing steps.

Lehe et al[14] developed a standardized scorecard for POC tests which suggested that having results printed, displayed and stored on the device was important; however, these were not ranked as an essential criterion for a POC device. Consensus was achieved from our Delphi survey for results from the point-of-care test to be automatically added remotely to patients’ records as an optional feature. Within the scorecard created by Lehe et al[14] wireless network connectivity was ranked as important, but not essential. In contrast, consensus was not achieved in our study for results to be sent via email to healthcare professionals, as the panel commented that this was not a required feature.

Following two rounds of the Delphi process, consensus was not achieved for criteria associated with costs of the POC test. Respondents expressed concern about the magnitude of costs, who would be responsible for costs, and if any perceived benefits of tests (e.g. faster notification of results) would outweigh a perception of greater cost. Similar concerns were raised in a study highlighting GP views on POC tests to identify common infections.[19] Kaman et al[13] reported that perceptions around costs associated with the POC tests for infectious diseases was found not to be a high priority for either medical personnel, manufacturers or the general public.[13] However, comments from this Delphi panel for these criteria suggested that it could be a useful alternative to current culture methods, especially if it was available through the National Health Service (NHS). Howick et al[19] also suggested that uptake of a POC test within primary care will be dependent on the type of reimbursement available and that further research is needed on this issue.[19] The panel also suggested that if the identification or lack of identification (i.e. a negative result) of the pathogen was achieved in a shorter timeframe, patients could receive the most appropriate treatment. This clearly has implications for both cost control and improved antibiotic stewardship, as well as the potential for improved patient care.

### Strengths and limitations

This study has several key strengths. The panel which completed the Delphi were from diverse geographical areas with a range of expertise and experience. The original criteria were developed using a number of reputable sources in addition to input from the Steering Group. The number of rounds, consensus method and limits for acceptance/rejection/revision were confirmed before the questionnaire was distributed. Furthermore, experts were given the opportunity to consider their level of agreement in Round 1 compared to the group response for the statements where consensus was not achieved.[23]

There were also limitations. The ASSURED guidelines were consulted in the development of the initial criteria; these are generally used in the context of the developing world, while the aim of our study was to develop a set of criteria for use in primary care in the developed world. However, we also referred to other resources such as the peer-reviewed scientific literature, clinical and laboratory guidelines, manufacturers’ literature, regulatory authorities and knowledge and experience from within the Steering Group. As with all Delphi studies, there is a lack of reproducibility as results depend on the experts included within the panel, and indeed the panel members had been identified by members of the Steering Group. It was our aim to receive feedback from as many stakeholders as possible. Thus, we selected a heterogeneous panel to ensure diverse ranges of opinions were obtained. As the responses provided by the panellists represented their own personal view, based on their experience, it can lead to bias, however, 17 experts completed the first round, and for Delphi studies, it has been recommended that to achieve optimal consensus, the panel should be between 5–15.[16]^,^[17] The number of responders declined in the second round [15 (88%) completed the survey], reflecting response rates noted in other Delphi studies.[16]^,^[24]

## Conclusions

Within our study, key themes have been discussed and agreed by the Delphi panel, indicating the important features of a POC test. Criteria relating to intended use, detection and identification of urinary pathogens, features and performance and ease of use, all achieved consensus. If such tests are to be used in everyday practice, more targeted treatment of antibiotics could be provided to patients for the treatment of UTIs. This study supports previous research in that the need for a POC UTI test has been highlighted [20]. We have also refined the criteria, with consensus on the type and characteristics of the test, across a broad spectrum of healthcare professionals.

However, there was lack of consensus when criteria relating to POC test costs were presented. This may be due, in part, to the current UK primary care system which involves sending samples for testing to central laboratories, and paying for this service from contract costs. Therefore, costs associated with current UTI testing may be less obvious. Longer term benefits could result in avoiding the need for further prescriptions of antibiotics resulting in additional cost saving. With growing concerns regarding antibiotic resistance, this topic is rapidly gaining importance. As Howick et al[19] suggested, there is a need for wider debate on POC test costs and a detailed health economic appraisal of the benefits of POC UTI testing, for the patient and for the healthcare providers.

## Supporting information

S1 Table Delphi panel responses from Round 1. (DOCX)

S2 Table Delphi panel responses from Round 2. (DOCX)

S1 File Round 1 questionnaire. (DOCX)

S2 File Round 2 questionnaire. (DOCX)

S3 File Round 1 comments from panel members. (DOCX)

S4 File Round 2 comments from panel members. (DOCX)
