# Supplementary material for: Criteria required for an acceptable point-of-care test for UTI detection: Obtaining consensus using the Delphi technique
Source: PLoS One. 2018 Jun 7;13(6):e0198595. doi: 10.1371/journal.pone.0198595 (PMC5991694; doi:10.1371/journal.pone.0198595)
Supplement: S2 Table — (DOCX) [file pone.0198595.s002.docx]

**Supporting Information Table 2: Delphi panel responses from Round 2.**

| **Statement Number** | **Delphi Panel Member** | | | | | | | | | | | | | | |
| --- | --- | --- | --- | --- | --- | --- | --- | --- | --- | --- | --- | --- | --- | --- | --- |
|  | **1** | **2** | **3** | **4** | **5** | **6** | **7** | **8** | **9** | **10** | **11** | **12** | **13** | **14** | **15** |
| **1** | A | A | A | N | A | A | A | A | A | A | N | N | A | A | SA |
|  | 4 | 4 | 4 | 3 | 4 | 4 | 4 | 4 | 4 | 4 | 3 | 3 | 4 | 4 | 5 |
| **2** | A | A | SA | N | A | N | SA | A | A | A | A | SD | N | SA | SA |
|  | 4 | 4 | 5 | 3 | 4 | 3 | 5 | 4 | 4 | 4 | 4 | 1 | 3 | 5 | 5 |
| **3** | A | A | A | A | A | A | A | A | A | D | A | N | A | A | A |
|  | 4 | 4 | 4 | 4 | 4 | 4 | 4 | 4 | 4 | 2 | 4 | 3 | 4 | 4 | 4 |
| **4** | A | A | A | A | SA | A | A | A | SA | A | A | A | D | A | A |
|  | 4 | 4 | 4 | 4 | 5 | 4 | 4 | 4 | 5 | 4 | 4 | 4 | 2 | 4 | 4 |
| **5** | A | A | A | A | D | A | A | D | N | A | A | A | D | A | SA |
|  | 4 | 4 | 4 | 4 | 2 | 4 | 4 | 2 | 3 | 4 | 4 | 4 | 2 | 4 | 5 |
| **6** | A | A | A | A | A | A | D | A | A | SA | SA | A | A | A | A |
|  | 4 | 4 | 4 | 4 | 4 | 4 | 2 | 4 | 4 | 5 | 5 | 4 | 4 | 4 | 4 |
| **7** | A | SD | A | N | A | N | A | N | N | D | A | A | SD | N | A |
|  | 4 | 1 | 4 | 3 | 4 | 3 | 4 | 3 | 3 | 2 | 4 | 4 | 1 | 3 | 4 |
| **8** | N | SD | A | SD | N | N | N | N | N | A | A | A | SD | N | SA |
|  | 3 | 1 | 4 | 1 | 3 | 3 | 3 | 3 | 3 | 4 | 4 | 4 | 1 | 3 | 5 |
| **9** | D | N | N | D | D | D | N | A | D | D | A | A | SD | N | A |
|  | 2 | 3 | 3 | 2 | 2 | 2 | 3 | 4 | 2 | 2 | 4 | 4 | 1 | 3 | 4 |
| **10** | A | D | A | N | D | N | A | N | A | D | SA | A | SD | A | A |
|  | 4 | 2 | 4 | 3 | 2 | 3 | 4 | 3 | 4 | 2 | 5 | 4 | 1 | 4 | 4 |
| **KEY:** SD- Strongly Disagree, D-Disagree, N-Neutral, A-Agree, SA- Strongly agree | | | | | | | | | | | | | | | |
