# Supplementary material for: Criteria required for an acceptable point-of-care test for UTI detection: Obtaining consensus using the Delphi technique
Source: PLoS One. 2018 Jun 7;13(6):e0198595. doi: 10.1371/journal.pone.0198595 (PMC5991694; doi:10.1371/journal.pone.0198595)
Supplement: S1 File — (DOCX) [file pone.0198595.s003.docx]

# Supporting Information File 1: Round 1 Questionnaire

**Section 1: Intended use of the point-of-care test.**

| **1. The point-of-care test can be used for any patient suspected of having a UTI (i.e. presenting with symptoms, such as confusion, agitation, concentrated urine, dehydration) regardless of age/demographic.** | | | | |
| --- | --- | --- | --- | --- |
| 1:Strongly disagree | 2:Disagree | 3:Uncertain | 4:Agree | 5:Strongly agree |
| Comment: | | | | |

| **2. The point-of-care test can be used within GP surgeries.**  Explanation: Urine samples are often received at GP surgeries. Therefore, the point-of-care test will be sufficiently easy to use with minimal training requirements. | | | | |
| --- | --- | --- | --- | --- |
| 1:Strongly disagree | 2:Disagree | 3:Uncertain | 4:Agree | 5:Strongly agree |
| Comment: | | | | |

| **3. The point-of-care test can be used within care home environments.**  Explanation: UTIs are common within care home environments, this leads to a significant number of urine samples. The point-of-care test will be sufficiently easy to use with minimal training requirements. | | | | |
| --- | --- | --- | --- | --- |
| 1:Strongly disagree | 2:Disagree | 3:Uncertain | 4:Agree | 5:Strongly agree |
| Comment: | | | | |

| **4. The point-of-care test can be used within secondary care for detection of urinary pathogens.** | | | | |
| --- | --- | --- | --- | --- |
| 1:Strongly disagree | 2:Disagree | 3:Uncertain | 4:Agree | 5:Strongly agree |
| Comment: | | | | |

| **5. The point-of-care test will require patients’ consent for their urine specimen.** | | | | |
| --- | --- | --- | --- | --- |
| 1:Strongly disagree | 2:Disagree | 3:Uncertain | 4:Agree | 5:Strongly agree |
| Comment: | | | | |

**Section 2: The detection and identification of potential urinary pathogens.**

| **6. Detection of the most common urinary pathogens will be achieved by the point-of-care test.**  Explanation: Urinary pathogens for detection by the point-of-care test include: *Escherichia coli, Klebsiella pneumoniae, Klebsiella oxytoca, Enterococcus faecalis, Enterococcus faecium, Proteus mirabilis, Proteus vulgaris, Proteus penneri, Providencia stuartii, Providencia rettgeri, Morganella morganii, Staphylococcus saprophyticus, Pseudomonas aeruginosa, Candida albicans, Staphylococcus aureus, Enterobacter cloacae, Enterobacter aerogenes, Serratia marcescens, Citrobacter koseri, Citrobacter freundii, Acinetobacter baumannii* and *Staphylococcus epidermidis.*  Urinary pathogens for detection were chosen based on clinical and laboratory guidelines as shown in Appendix 2.2. | | | | |
| --- | --- | --- | --- | --- |
| 1:Strongly disagree | 2:Disagree | 3:Uncertain | 4:Agree | 5:Strongly agree |
| Comment: | | | | |

| **7. Results obtained by the point-of-care test should have a high sensitivity.**  Explanation: High probability of correctly detecting and identifying a urinary pathogen. | | | | |
| --- | --- | --- | --- | --- |
| 1:Strongly disagree | 2:Disagree | 3:Uncertain | 4:Agree | 5:Strongly agree |
| Comment: | | | | |

| **8. Results obtained by the point-of-care test should have a high specificity.**  Explanation: High probability of accurately identifying the absence of a urinary pathogen. | | | | |
| --- | --- | --- | --- | --- |
| 1:Strongly disagree | 2:Disagree | 3:Uncertain | 4:Agree | 5:Strongly agree |
| Comment: | | | | |

| **9. Results obtained by the point-of-care test should have a high positive predictive value.**  Explanation: Positive predictive value – probability that those samples which test positive with the point-of-care test truly have a urinary pathogen present. | | | | |
| --- | --- | --- | --- | --- |
| 1:Strongly disagree | 2:Disagree | 3:Uncertain | 4:Agree | 5:Strongly agree |
| Comment: | | | | |

| **10. Results obtained by the point-of-care test should have a high negative predictive value.**  Explanation: Negative predictive value – probability that those samples which do not test positive with the point of-care test truly do not have a urinary pathogen present. | | | | |
| --- | --- | --- | --- | --- |
| 1:Strongly disagree | 2:Disagree | 3:Uncertain | 4:Agree | 5:Strongly agree |
| Comment: | | | | |

| **11. The level of detection required by the point-of-care test for the urinary pathogens is between 10^2^-10^5^ CFU/ml.**  Explanation: Based on: 1. Clinical guidelines including Scottish Intercollegiate Guidelines (SIGN), European Association of Urology (EAU), and Infectious Disease Society of America (IDSA). 2. Laboratory guidelines including Cumulative techniques and procedures in clinical microbiology (Cumitech) and Public Health England.  See Appendix 2.3 for a summary of the level of detection suggested by these groups to indicate presence of urinary pathogens. | | | | |
| --- | --- | --- | --- | --- |
| 1:Strongly disagree | 2:Disagree | 3:Uncertain | 4:Agree | 5:Strongly agree |
| Comment: | | | | |

| **12. Identification of urinary pathogens should be to the genus or species level as appropriate.**  Explanation: Identification of urinary pathogens will be at the same level or better than determined by conventional culture. Current culture methods do not typically identify non- *E. coli* *Enterobacteriaceae* to a more detailed level (e.g. genus or species) unless multi-resistance is present. However, the point-of-care test will improve this level of identification by reporting *Enterobacteriaceae* at the genus level, e.g. *Klebsiella spp*., *Citrobacter spp*., *Enterobacter spp*. or *Proteus spp*. or to species level e.g. *Morganella morganii*, *Acinetobacter baumannii.* | | | | |
| --- | --- | --- | --- | --- |
| 1:Strongly disagree | 2:Disagree | 3:Uncertain | 4:Agree | 5:Strongly agree |
| Comment: | | | | |

| **13. Appropriate prescribing of antibiotics will be aided by the point-of-care test.**  Explanation: Identification of urinary pathogens will be achieved in less than 4 hours. This would permit the prescription of an appropriate antibiotic by the GP, which could then be dispensed to the patient within 24 hours of the specimen being received. Currently if the urine sample is sent for culture, this typically is achieved within 3-4 days. | | | | |
| --- | --- | --- | --- | --- |
| 1:Strongly disagree | 2:Disagree | 3:Uncertain | 4:Agree | 5:Strongly agree |
| Comment: | | | | |

**Section 3: Features and performance of the point-of-care device.**

| **14. Detection and identification of pathogens directly from the urine sample will be completed in a one-step process.**  Explanation: Once a sample has been loaded onto the instrument, no further input is required from the staff member. | | | | |
| --- | --- | --- | --- | --- |
| 1:Strongly disagree | 2:Disagree | 3:Uncertain | 4:Agree | 5:Strongly agree |
| Comment: | | | | |

| **15. The point-of-care test should offer quicker detection than conventional culture methods to better inform clinical decision making.**  Explanation: Detection and identification of urinary pathogens will be completed within a more clinically relevant time frame. | | | | |
| --- | --- | --- | --- | --- |
| 1:Strongly disagree | 2:Disagree | 3:Uncertain | 4:Agree | 5:Strongly agree |
| Comment: | | | | |

| **16. The point-of-care test should operate as a stand-alone instrument.**  Explanation: Consumables for the point-of-care test will include: urine collection pot, disposable pipette, test reagent cartridge and the instrument. | | | | |
| --- | --- | --- | --- | --- |
| 1:Strongly disagree | 2:Disagree | 3:Uncertain | 4:Agree | 5:Strongly agree |
| Comment: | | | | |

| **17. A small sample volume of urine will be required for the point-of-care test.**  Explanation: The test will use approximately 1 ml of mid-stream urine. Mid-stream urine will be used to minimise contaminating flora. | | | | |
| --- | --- | --- | --- | --- |
| 1:Strongly disagree | 2:Disagree | 3:Uncertain | 4:Agree | 5:Strongly agree |
| Comment: | | | | |

| **18. Only one sample will be analysed at a time, using the point-of-care test.** | | | | |
| --- | --- | --- | --- | --- |
| 1:Strongly disagree | 2:Disagree | 3:Uncertain | 4:Agree | 5:Strongly agree |
| Comment: | | | | |

| **19. The space required for the point-of-care test instrument and operation should be minimal.**  Explanation: Size of the instrument will be approximately 30cm x 60cm x 45cm (Width x Depth x Height). | | | | |
| --- | --- | --- | --- | --- |
| 1:Strongly disagree | 2:Disagree | 3:Uncertain | 4:Agree | 5:Strongly agree |
| Comment: | | | | |

| **20. As part of the point-of-care test, unique barcode sample tracking will be provided.**  Explanation: Samples will be identified using unique barcodes containing information such as patient name, date of birth, gender, patient identification number, name of general practitioner and date of sampling. | | | | |
| --- | --- | --- | --- | --- |
| 1:Strongly disagree | 2:Disagree | 3:Uncertain | 4:Agree | 5:Strongly agree |
| Comment: | | | | |

| **21. Results can be stored on the point-of-care test.**  Explanation: Results will be stored on the point-of-care test as a backup and for audit purposes. | | | | |
| --- | --- | --- | --- | --- |
| 1:Strongly disagree | 2:Disagree | 3:Uncertain | 4:Agree | 5:Strongly agree |
| Comment: | | | | |

| **22. Results can be printed directly from the point-of-care test.**  Explanation: A hard copy of the results can be added to the patient’s clinical records. | | | | |
| --- | --- | --- | --- | --- |
| 1:Strongly disagree | 2:Disagree | 3:Uncertain | 4:Agree | 5:Strongly agree |
| Comment: | | | | |

| **23. Results from the point-of-care test can be automatically added remotely to patient’s records as an optional feature.**  Explanation: Optional because this will incur extra costs to cover integration of networking capabilities. | | | | |
| --- | --- | --- | --- | --- |
| 1:Strongly disagree | 2:Disagree | 3:Uncertain | 4:Agree | 5:Strongly agree |
| Comment: | | | | |

| **24. Relevant healthcare professionals can be notified of results from the point-of-care test automatically via email as an optional feature.**  Explanation: Optional because this will incur extra costs to cover integration of networking capabilities. For this to be achieved clinical governance frameworks would need to be considered. | | | | |
| --- | --- | --- | --- | --- |
| 1:Strongly disagree | 2:Disagree | 3:Uncertain | 4:Agree | 5:Strongly agree |
| Comment: | | | | |

**Section 4: Operation of the point-of-care test by user.**

| **25. Minimal staff training should be required to use the point-of-care test.**  Explanation: Staff training will be provided to ensure accuracy and familiarity for the use of the instrument. | | | | |
| --- | --- | --- | --- | --- |
| 1:Strongly disagree | 2:Disagree | 3:Uncertain | 4:Agree | 5:Strongly agree |
| Comment: | | | | |

| **26. Staff operation of the point-of-care test will include the following steps:**   1. **Collection of urine sample from the patient.** | | | | |
| --- | --- | --- | --- | --- |
| 1:Strongly disagree | 2:Disagree | 3:Uncertain | 4:Agree | 5:Strongly agree |
| Comment: | | | | |

| 1. **Storage of the urine sample if required.**   Explanation: Storage of the urine sample will only be required if there is a backlog of samples to be analysed. | | | | |
| --- | --- | --- | --- | --- |
| 1:Strongly disagree | 2:Disagree | 3:Uncertain | 4:Agree | 5:Strongly agree |
| Comment: | | | | |

| 1. **Safe handling and loading of the urine sample onto the instrument.** | | | | |
| --- | --- | --- | --- | --- |
| 1:Strongly disagree | 2:Disagree | 3:Uncertain | 4:Agree | 5:Strongly agree |
| Comment: | | | | |

| 1. **Input of sample information via the touch screen on the instrument.** | | | | |
| --- | --- | --- | --- | --- |
| 1:Strongly disagree | 2:Disagree | 3:Uncertain | 4:Agree | 5:Strongly agree |
| Comment: | | | | |

| 1. **Safe disposal of the urine sample.** | | | | |
| --- | --- | --- | --- | --- |
| 1:Strongly disagree | 2:Disagree | 3:Uncertain | 4:Agree | 5:Strongly agree |
| Comment: | | | | |

| **27. Maintenance and quality control will be required for the point-of-care instrument.**  Explanation: Training will be provided for maintenance of the instrument and quality control procedures. | | | | |
| --- | --- | --- | --- | --- |
| 1:Strongly disagree | 2:Disagree | 3:Uncertain | 4:Agree | 5:Strongly agree |
| Comment: | | | | |

| **28. The time required by staff (as detailed in statements 26a-e) to run the point-of-care test will be minimal.**  Explanation: Staff time will be required for training, maintenance of equipment, quality control of the instrument and recording of the results. | | | | |
| --- | --- | --- | --- | --- |
| 1:Strongly disagree | 2:Disagree | 3:Uncertain | 4:Agree | 5:Strongly agree |
| Comment: | | | | |

**Section 5: Costs associated with the point-of-care test.**

| **29. Would you be willing to pay £30 (€38) per sample to detect and identify the most common urinary pathogens within 4 hours (cost includes the price of the point-of-care instrument)?**  Explanation: By comparison conventional culture methods cost around £35 (€44.45) per sample and typically take 48-120 hours for detection and identification of pathogens (cost excludes staff wages). | | | | |
| --- | --- | --- | --- | --- |
| 1:Strongly disagree | 2:Disagree | 3:Uncertain | 4:Agree | 5:Strongly agree |
| Comment: | | | | |

| **30. If the initial device cost less than £10,000 (€12,403) would you be interested in buying the device?** | | | | |
| --- | --- | --- | --- | --- |
| 1:Strongly disagree | 2:Disagree | 3:Uncertain | 4:Agree | 5:Strongly agree |
| Comment: | | | | |

| **31. If network capabilities would incur an additional cost of £5 (€6.30) per sample, would you be willing to pay for this feature?**  Explanation: Network capabilities would permit result notification by email and/or remote result storage (e.g. automatic update of patient records.) | | | | |
| --- | --- | --- | --- | --- |
| 1:Strongly disagree | 2:Disagree | 3:Uncertain | 4:Agree | 5:Strongly agree |
| Comment: | | | | |

| **32. Would you be willing to pay an extra £5 (€6.30) per sample to achieve the test result in <2hours?** | | | | |
| --- | --- | --- | --- | --- |
| 1:Strongly disagree | 2:Disagree | 3:Uncertain | 4:Agree | 5:Strongly agree |
| Comment: | | | | |

| **33. Would you be willing to pay an extra £2.50 (€3.10) per sample to detect genetic indicators of resistance to trimethoprim?** | | | | |
| --- | --- | --- | --- | --- |
| 1:Strongly disagree | 2:Disagree | 3:Uncertain | 4:Agree | 5:Strongly agree |
| Comment: | | | | |
