# Supplementary material for: Criteria required for an acceptable point-of-care test for UTI detection: Obtaining consensus using the Delphi technique
Source: PLoS One. 2018 Jun 7;13(6):e0198595. doi: 10.1371/journal.pone.0198595 (PMC5991694; doi:10.1371/journal.pone.0198595)
Supplement: S2 File — (DOCX) [file pone.0198595.s004.docx]

**Supporting Information File 2: Round 2 Questionnaire**

**Section 1: Intended use of the point-of-care test.**

| **1. The POC test can be used within secondary care as a “one-step” test for the detection and identification of urinary pathogens when a faster result is required.**  **Explanation:** In comparison to a) Matrix-assisted laser desorption/ionization time-of-flight (MALDI-TOF) which identifies pathogens once detected and b) the dipstick which only detects the presence of a pathogen, this POC test will detect and identify the urinary pathogens in a one-step process. | | | | |
| --- | --- | --- | --- | --- |
| 1:Strongly disagree | 2:Disagree | 3:Uncertain | 4:Agree | 5:Strongly agree |
| Comment: | | | | |

| **2. The POC test will require patients’ consent (where possible and practicable) for their urine specimen as occurs with all diagnostic tests.** | | | | |
| --- | --- | --- | --- | --- |
| 1:Strongly disagree | 2:Disagree | 3:Uncertain | 4:Agree | 5:Strongly agree |
| Comment: | | | | |

**Section 2: The detection and identification of potential urinary pathogens.**

| **3. The level of detection required by the POC test for the urinary pathogens is between 10^2^-10^5^ CFU/ml as determined by the published guidelines.**  ***Explanation:*** Based on: 1. Clinical guidelines including Scottish Intercollegiate Guidelines (SIGN), European Association of Urology (EAU), and Infectious Disease Society of America (IDSA). 2. Laboratory guidelines including Cumulative techniques and procedures in clinical microbiology (Cumitech) and Public Health England. See Appendix 2.3 for a summary of the level of detection suggested by these groups to indicate presence of urinary pathogens. | | | | |
| --- | --- | --- | --- | --- |
| 1:Strongly disagree | 2:Disagree | 3:Uncertain | 4:Agree | 5:Strongly agree |
| Comment: | | | | |

**Section 3: Features and performance of the POC device.**

| **4. Only one sample will be analysed at a time, using the point-of-care test.** | | | | |
| --- | --- | --- | --- | --- |
| 1:Strongly disagree | 2:Disagree | 3:Uncertain | 4:Agree | 5:Strongly agree |
| Comment: | | | | |

| **5. Relevant healthcare professionals can be notified of results from the POC test automatically via email as an optional feature.**  **Explanation:** Optional because this will incur extra costs to cover integration of networking capabilities. For this to be achieved clinical governance frameworks (for example, data security and confidentiality) would need to be considered. | | | | |
| --- | --- | --- | --- | --- |
| 1:Strongly disagree | 2:Disagree | 3:Uncertain | 4:Agree | 5:Strongly agree |
| Comment: | | | | |

**Section 4: Operation of the POC test by user.**

| **6. Staff operation of the POC test will include:**  **-Receipt and verification of the urine sample from the patient.** | | | | |
| --- | --- | --- | --- | --- |
| 1:Strongly disagree | 2:Disagree | 3:Uncertain | 4:Agree | 5:Strongly agree |
| Comment: | | | | |

**Section 5: Costs associated with the POC test.**

| **7. Would you be willing to pay £30 (€38) per sample to detect and identify the most common urinary pathogens within 4 hours (cost includes the price of the point-of-care instrument)?**  Explanation: By comparison conventional culture methods cost around £35 (€44.45) per sample and typically take 48-120 hours for detection and identification of pathogens (cost excludes staff wages). | | | | |
| --- | --- | --- | --- | --- |
| 1:Strongly disagree | 2:Disagree | 3:Uncertain | 4:Agree | 5:Strongly agree |
| Comment: | | | | |

| **8. If the initial device cost less than £10,000 (€12,403) would you be interested in buying the device?** | | | | |
| --- | --- | --- | --- | --- |
| 1:Strongly disagree | 2:Disagree | 3:Uncertain | 4:Agree | 5:Strongly agree |
| Comment: | | | | |

| **9. Would you be willing to pay an extra £5 (€6.30) per sample to achieve the test result in <2hours?** | | | | |
| --- | --- | --- | --- | --- |
| 1:Strongly disagree | 2:Disagree | 3:Uncertain | 4:Agree | 5:Strongly agree |
| Comment: | | | | |

| **10. Would you be willing to pay an extra £2.50 (€3.10) per sample to detect genetic indicators of resistance to trimethoprim which may better inform appropriate antibiotic treatment?** | | | | |
| --- | --- | --- | --- | --- |
| 1:Strongly disagree | 2:Disagree | 3:Uncertain | 4:Agree | 5:Strongly agree |
| Comment: | | | | |
