# Supplementary material for: Criteria required for an acceptable point-of-care test for UTI detection: Obtaining consensus using the Delphi technique
Source: PLoS One. 2018 Jun 7;13(6):e0198595. doi: 10.1371/journal.pone.0198595 (PMC5991694; doi:10.1371/journal.pone.0198595)
Supplement: S3 File — (DOCX) [file pone.0198595.s005.docx]

# Supporting Information File 3: Round 1 comments from panel members.

Please note all comments are verbatim.

**Section 1: Intended use of the point-of-care test.**

1. **The point-of-care test can be used for any patient suspected of having a UTI (i.e. presenting with symptoms, such as confusion, agitation, concentrated urine, dehydration) regardless of age/demographic.**

Comments:

Hard to answer this with confidence without seeing the technical specifications and protocol for the test! POC testing by trained staff in a hospital setting should be feasible, depending on the test.

Use of other or space for own comment

Surely, this is the only acceptable answer.

A proportion of older patients will have pre-existing bacteriuria and using a POC test may lead to a false positive for UTI in a patient with non-specific symptoms.

A POC test that can only be validated for use on a subset of patients with possible UTI may still be of value if it can reliably rule out/rule in UTI within a specific cohort.

early detection can alter length of stay in any patient group

There are a wide variety of patients with UTI, and it may be appropriate to target, as the urine findings will be different in infections in different groups. For instance in pregnant women any bacteria in the urine (bacteruria) is significant, whereas in catheterised patients bacteruiria may be entirely incidental.

may not be appropriate for some depending on the validity/accuracy of the "point of care" test.

Would help prevent prescription of innaproriate antibiotics in many patients

Clinical context is important and other aspects of history and examination would be important.

1. **The point-of-care test can be used within GP surgeries.**

Explanation: Urine samples are often received at GP surgeries. Therefore, the point-of-care test will be sufficiently easy to use with minimal training requirements.

Comments:

Hard to answer this with confidence without seeing the technical specifications and protocol for the test! POC testing by trained staff in a hospital setting should be feasible, depending on the test.

admission avoidance

This is desirable. Consideration should be given to inter-user variability analysis between different professional groups when validating the test- there is evidence that point of care tests done by biomedical scientist staff in laboratories perform significantly differently versus other non laboratory staff.

Would reduce pressure on Gp Surgeries and labs and would help with accurate prescriptions

But not exclusively

1. **The point-of-care test can be used within care home environments.**

Explanation: UTIs are common within care home environments, this leads to a significant number of urine samples. The point-of-care test will be sufficiently easy to use with minimal training requirements.

Comments:

This assumes use by care staff who have possibly had minimal or no training...

How will this reduce the need to culture the organsim?

Caveats from Q1 notwithstanding (i.e. older individuals likely to have chronic bacteriuria).

I think we would call these nursing homes or long term care facilities in the US.

I agree if the POC test is simple to perform

See answer 4 above. There is no reason to suspect that patients would be less reliable testers of their own urine that healthcare professionals

Dont feel the control measures would be in place in care homes to provide assurances that the test had been used correctly

1. **The point-of-care test can be used within secondary care for detection of urinary pathogens.**

Comments:

Comments as above

Yes.

Not so critical with laboratory on site and new MALDI-TOF technology for rapid identification. Difficulty remains with older patients who have chronic bacteriuria. What does POC test add to dipstick?

Most patients with UTI are treated in primary care, and the need of POC here is highest. Secondary care has a number of other tests available.

Would reduce pressure on labs

1. **The point-of-care test will require patients’ consent for their urine specimen.**

Comments:

Specific consent is generally not required for routine diagnostic testing (assumes the test is CE-IVD approved, supported by accredited testing lab, and not a research project!)

Assume consent when trying to investigate or diagnose

You are supposed to get verbal consent for all patients capable of giving consent regardless of what you are doing. Otherwise it is battery.

Should be treated the same as any diagnostic test.

I am not sure why this is an issue about the performance of a POC. It would seem to me that the patient would give consent a priori as terms of their care.

If the test is validated, I don't see that additional specific consent would be required (over and above implied consent for medical investigations)

non invasive proceedure

All interventions and investigations should require consent

where appropriate i.e pt may be confused and unable to give valid consent

Gaining consent from someone who is acutely confused or has dementia is going to be impossible in this context. In this case, practically, the autonomy of the individual is overridden taking the ethical principles of beneficence and non-malificence as having primacy.

**Section 2: The detection and identification of potential urinary pathogens.**

1. **Detection of the most common urinary pathogens will be achieved by the point-of-care test.**

Explanation: Urinary pathogens for detection by the point-of-care test include: *Escherichia coli, Klebsiella pneumoniae, Klebsiella oxytoca, Enterococcus faecalis, Enterococcus faecium, Proteus mirabilis, Proteus vulgaris, Proteus penneri, Providencia stuartii, Providencia rettgeri, Morganella morganii, Staphylococcus saprophyticus, Pseudomonas aeruginosa, Candida albicans, Staphylococcus aureus, Enterobacter cloacae, Enterobacter aerogenes, Serratia marcescens, Citrobacter koseri, Citrobacter freundii, Acinetobacter baumannii* and *Staphylococcus epidermidis.* Urinary pathogens for detection were chosen based on clinical and laboratory guidelines as shown in Appendix 2.2

Comments:

Not sure there is need for so many pathogens to diagnose a uti! And treat appropriately?

Good coverage of common pathogens.

Some of these organisms such as S. epidermidis are likely to be frequent urine contaminants. Otherwise these organisms seem reasonable. One of the tricky organisms is Streptococcus agalactiae. Detection of it in asymptomatic carriage in pregnant women is important to detect. How to include this in test would be of interest.

The spectrum of bacteria mentioned cover the vast majority of all UTIs

Streptococcus agalactiae is omitted from the list in question 11, this significant organism should be detectable in this system.

1. **Results obtained by the point-of-care test should have a high sensitivity.**

Explanation: High probability of correctly detecting and identifying a urinary pathogen.

Comments:

Will obviously help to treat effectively on first antibiotics

Surely this would be the standard to be achieved.

If you wish to use in primary care or home environment unless sensitivity and specificity are very high test will be useless

Reliability

1. **Results obtained by the point-of-care test should have a high specificity.**

Explanation: High probability of accurately identifying the absence of a urinary pathogen.

Comments:

Otherwise waste of time and inappropriate use of meds

Surely this would be the standard to be achieved.

Over-treatment of suspected UTI is a significant challenge for antibiotic stewardship, particularly in older individuals.

If you were to have a good screening test one want a test with high sensitivity and high negative predictive value since most test will be negative. Want to screen them out.

If you wish to use in primary care or home environment unless sensitivity and specificity are very high test will be useless

This may be a more important feature than sensitivity since, in practice, UTI is often over-diagnosed

Not correct interpretation in the text! If you want to rule out a UTI you should go for a test with a high sensitivity (Sensitivity-Negative-OUT), SNOUT)

Reliability

1. **Results obtained by the point-of-care test should have a high positive predictive value.** Explanation: Positive predictive value – probability that those samples which test positive with the point-of-care test truly have a urinary pathogen present.

Comments:

Surely this would be the standard to be achieved.

I think high PPV is very important. The current point of care testing for UTI is dipstick testing which has a high NPV but a poor PPV. Despite this the dipstick is often interpreted as having a high PPV. So a new and improved system should provide a high positive predictive value.

Accuracy

1. **Results obtained by the point-of-care test should have a high negative predictive value.** Explanation: Negative predicative value – probability that those samples which do not test positive with the point of-care test truly do not have a urinary pathogen present.

Comments:

Surely this would be the standard to be achieved.

If you were to have a good screening test one want a test with high sensitivity and high negative predictive value since most test will be negative. Want to screen them out.

1. **The level of detection required by the point-of-care test for the urinary pathogens is between 10^2^-10^5^ CFU/ml.**

Explanation: Based on: 1. Clinical guidelines including Scottish Intercollegiate Guidelines (SIGN), European Association of Urology (EAU), and Infectious Disease Society of America (IDSA). 2. Laboratory guidelines including Cumulative techniques and procedures in clinical microbiology (Cumitech) and Public Health England. Please see Appendix 2.3 for a summary of the level of detection suggested by these groups to indicate presence of urinary pathogens.

Comments:

I favour a lower cut-off of 10^3.

Think the lower level of detection is too low. 1000 or even 10,000 is fine as the lowest level of detection.

The question is where to set the cutt of point with so many different recommandations

I agree, but the differences in the table above highlight the challenge in getting a single platform which would be equally useful in all patient groups.

microbiology specialist input would be best to answer this question

1. **Identification of urinary pathogens should be to the genus or species level as appropriate.** Explanation: Identification of urinary pathogens will be at the same level or better than determined by conventional culture. Current culture methods do not typically identify non- *E. coli* *Enterobacteriaceae* to a more detailed level (e.g. genus or species) unless multi-resistance is present. However, the point-of-care test will improve this level of identification by reporting *Enterobacteriaceae* at the genus level, e.g. *Klebsiella spp*., *Citrobacter spp*., *Enterobacter spp*. or *Proteus spp*. or to species level e.g. *Morganella morganii*, *Acinetobacter baumannii.*

Comments:

Will be useful for patients with recurrent Utis and repeated use of antibiotics and hosp admissions

Is there a point to identify this accurately? It won't affect the management. Seems academic.

Failure to speciate coliforms is a source of frustration when attempting to monitor resistance trends.

I think this is more useful for epidemiological purposes rather than for the clinical benefit of the individual patient.

1. **Appropriate prescribing of antibiotics will be aided by the point-of-care test.** Explanation: Identification of urinary pathogens will be achieved in less than 4 hours. This would permit the prescription of an appropriate antibiotic by the GP, which could then be dispensed to the patient within 24 hours of the specimen being received. Currently if the urine sample is sent for culture, this typically is achieved within 3-4 days.

Comments:

Yes, a rapid result with a test that can effectively detect a broad range of bacterial pathogens would enable more focused Ab treatment (i.e. Gram +ve / Gram -ve agents, and guideline-based treatment for specific organisms). However, culture to obtain phenotypic sensitivity data may still be required...

Huge time diffenerence in time testing which will be highly effective patient wise and cost wise. Reduce need for hosp admissions if treated properly and promptly by gp

That would be great, but you can't really say what antibiotics the UTI will really be resistant to without culture.

The challenge we face is that patients with obvious symptoms of UTI usually warrant treatment but a group of patients with non-specific symptoms (usually older individuals) often has pre-existing bacteriuria so identifying an organism from urine is not necessarily helpful in determining the presence of a UTI and may legitimise prescribing of antibiotics to this group.

This could be based on organism identification, prior history and regional or local antibiograms.

Avoidance of antibiotic prescription, where no UTI is present, is also an important considertion

Particularly, a good POC test (with high sensitivity) can reduce inappropriate overprescribing by ruling out patients not to be treated with antibiotics

It is usually a core funding justification of such products that antibiotic prescribing will be improved. This is a potential benefit. However the interpretation of the test, including the pre-test assessment of which patients are tested have to be considered if improvements in antibtioic prescribing are to be realised. For instance if this test is used to screen catheterised patients, then bacteria will be detected, irrespective of the presence or absence of clinical infection. In the abscence of infection, such a test will merely speed up process of giving patients innapropriate antibiotics. Careful consideration should be given to the trails of the product. In order to convince those tasked with antibiotic stewardship that point of care tests will make a useful difference to prescribing trends, such prescribing trends should be included as primary trail outcomes.

**Section 3: Features and performance of the** **point-of-care device**

1. **Detection and identification of pathogens directly from the urine sample will be completed in a one-step process.**

Explanation: Once a sample has been loaded onto the instrument, no further input is required from the staff member.

Comments:

Can't comment on this without understanding of the test chemistry/protocol.

Ease of use and not time consuming

This will allow less trained individuals to use this device and reduce technical errors.

Each step is an additional potential error

1. **The point-of-care test should offer quicker detection than conventional culture methods to better inform clinical decision making.**

Explanation: Detection and identification of urinary pathogens will be completed within a more clinically relevant time frame.

Comments:

You probably still need to culture the organsim to be sure.

Quicker decision making is good. Accuracy is more important however. More intelligent decision making is more important still.

1. **The point-of-care test should operate as a stand-alone instrument.**

Explanation: Consumables for the point-of-care test will include: urine collection pot, disposable pipette, test reagent cartridge and the instrument.

Comments:

This would be essential for use in the clinical settings described above (GP surgery, care homes)

1. **A small sample volume of urine will be required for the point-of-care test.**

Explanation: The test will use approximately 1 ml of mid-stream urine. Mid-stream urine will be used to minimise contaminating flora.

Comments:

Seems small. Query will decrease yield.

Urine specimens are typically easy to obtains so specimen volume is not a limiting step usually.

Please factor into your thinking that many urine samples are not actually mid-stream. It is difficult for many patients to understand and execute a mid-stream sample. If your system validation rests heavily of the concept of the 'low contamination' mid stream sample i suggest you consider exclude groups such as children, catheterised patients, confused/ demented patients etc.

1. **Only one sample will be analysed at a time, using the point-of-care test.**

Comments:

It seems unlikely that POC testing of multiple samples would be required. Assuming a rapid test, then sequential testing would be possible.

Avoid mistakes

Will the whole process take 4 hours before you are allowed to test the next?

Depends on whether the equipment is unavailable for several hours while a single sample is processed.

In a busy clinic or emergency department that may be problematic but in most settings a single specimen being tested in reasonable as long as it takes only a matter of minutes. If it takes longer, then multiple samples will need to analyzed so random access would be desirable.

In a primary care or home care setting, testing a single sample at a time would be sufficient. In a secondary care setting, such as a paediatric emergency department, the ability to test multiple specimens simultaneously would be an advantage

Batching outside of a laboratory setting is error prone.

1. **The space required for the point-of-care test instrument and operation should be minimal.** Explanation: Size of the instrument will be approximately 30cm x 60cm x 45cm (Width x Depth x Height).

Comments:

Size of the footproint is always important.

Suggested size is excessive for use in the community

Clinical space in most healthcare areas is already compromised.

1. **As part of the point-of-care test, unique barcode sample tracking will be provided.** Explanation: Samples will be identified using unique barcodes containing information such as patient name, date of birth, gender, patient identification number, name of general practitioner and date of sampling.

Comments:

Probably not critical for POC testing

Time of sampling is also valuable.

Less likely to have mistakes in specimen identification since the majority of lab error occur at the pre- or post-analytical stages.

1. **Results can be stored on the point-of-care test.**

Explanation: Results will be stored on the point-of-care test as a backup and for audit purposes.

Comments:

Audit in into so stages extremely important and feedback from users

Handy

Makes retrieval of results easier

I would be interested in an option for the results to be uploaded in real-time via wifi seemlessly to the EHR

1. **Results can be printed directly from the point-of-care test.**

Explanation: A hard copy of the results can be added to the patient’s clinical records.

Comments:

Useful to print onto a label that can be put straight into the patient's notes.

Records in hosp or GP notes essential

Needs that function.

Useful to reduce risk of transcription error. Alternatively, e-mail result to clinician?

Should have a way to interface with electronic records. More efficient; less likely to have errors.

Any print-offs should be auditable on the devices software.

This would elimate errors with duplication

Yes in an ideal world it should interface seemlessly

1. **Results from the point-of-care test can be automatically added remotely to patient’s records as an optional feature.**

Explanation: Optional because this will incur extra costs to cover integration of networking capabilities.

Comments:

This raises many issues of data security etc.

Paper copy print out sufficient

Needs this.

Should have a way to interface with electronic records. More efficient; less likely to have errors.

Connectivity to electronic records is vital. Governance of point of care tests is often sub-optimal. They should all be under the supervision of an accredited laboratory.

1. **Relevant healthcare professionals can be notified of results from the point-of-care test automatically via email as an optional feature.**

Explanation: Optional because this will incur extra costs to cover integration of networking capabilities. For this to be achieved clinical governance frameworks would need to be considered.

Comments:

This raises many issues of data security etc.

Not necessary. Phone call or letter sufficient in early stages. Could be added in at later stage if deemed aporopriate and not too expensive

Would be great. Is there a question over confidentiality with this though?

Several challenges as relate to patient privacy but seems worthwhile.

**Section 4: Operation of the point-of-care test by user**

1. **Minimal staff training should be required to use the point-of-care test.**

Explanation: Staff training will be provided to ensure accuracy and familiarity for the use of the instrument.

Comments:

POC testing assumes use by non-specialist users, with appropriate training - not necessarily "minimal" training...

The less training, the more likely than it would be accepted.

See answer 4 above

Need to be able to cascade this training

**26. Staff operation of the point-of-care test will include the following steps:**

**a. Collection of urine sample from the patient.**

Comments:

Again, a choice needs to be made. If all samples are collected, or at least supervised by the staff operator the device may have a more limited range of patients

not sure what you mean by "collection". the patient would usually "collect" the sample and the staff would "receive and verify" the sample

Patient or their carer should ideally collect the sample

**b. Storage of the urine sample if required.**

Explanation: Storage of the urine sample will only be required if there is a backlog of samples to be analysed.

Comments:

This depends on the answer to question 51- if samples are being taken by patients in there own homes then there is an inevitable storage/ transit issue

**c. Safe handling and loading of the urine sample onto the instrument.**

No Comments

**d. Input of sample information via the touch screen on the instrument.**

Comments:

Much better if this could be done by scanning of barcodes on the specimen container.

Obviously minimal transcription steps are preferable. Transcription= error

Ideally scanned QR code or Bar Code

**e. Safe disposal of the urine sample.**

Comments:

Assume universal precautions in all setting wetter it is home GP or hosp

1. **Maintenance and quality control will be required for the point-of-care instrument.** Explanation: Training will be provided for maintenance of the instrument and quality control procedures.

Comments:

Existing POC tests in healthcare (i.e. clinical biochemistry) are managed by a specialist team who ensure maintenance is performed and QC performance is acceptable. It is reasonable to train users to perform routine (i.e. daily) maintenance.

Probably should be a technician available if needed.

Reason POC fail is because instrumentation is not maintained and quality control is ignored so this is very important

the task of maintaing and servicing should be with the supplying company

1. **The time required by staff (as detailed in statements 26a-e) to run the point-of-care test will be minimal.**

Explanation: Staff time will be required for training, maintenance of equipment, quality control of the instrument and recording of the results.

Comments:

Hands-on time is always a factor for healthcare staff with many other duties...

Should take 20 mins to explain as staff already trained to do a lot of this already.

Otherwise they will not want to do it.

This is essential for POC devices.

**Section 5: Costs associated with the point-of-care test**

1. **Would you be willing to pay £30 (€38) per sample to detect and identify the most common urinary pathogens within 4 hours (cost includes the price of the point-of-care instrument)?** Explanation: By comparison conventional culture methods cost around £35 (€44.45) per sample and typically take 48-120 hours for detection and identification of pathogens (cost excludes staff wages).

Comments:

Currently under block contract with hospital laboratory

Cover by medical card holders as elderly will not pay for this.

Probably saves money compared to culturing.

The cost differential of Â£5 is simply not worth the acquisition cost, or te oporunity cost of managing, of an inferior approach to assessment

The cost per test may need to be lower, given that at least a subset of urine samples from patients with UTI will still need to be sent for standard culture and antimicrobial susceptibility testing (in some cases to guide clinical management, but also for epidemiological purposes (such as determining local antibiograms to guide empiric therapy))

This price point is much too expensive for the current lower tech options to be dismissed

1. **If the initial device cost less than £10,000 (€12,403) would you be interested in buying the device?**

Comments:

Couldn't afford it if not provided for us

This type of instrument is generally placed on a reagent rental basis.

Hosp setting so it would be their decision in conjunction with micro and inf control etc.

I have no idea. What is this cost compared to the normal cost. .I have no frame of reference

Depends on how many specimens can be processed simultaneously.

this should be funded by the board.

Highly unlikely at this cost

1. **If network capabilities would incur an additional cost of £5 (€6.30) per sample, would you be willing to pay for this feature?** Explanation: Network capabilities would permit result notification by email and/or remote result storage (e.g. automatic update of patient records.)

Comments:

Expensive additional cost per sample

Data transfer is not this expensive.Is there a hidden cost here for setting up the secure data transfer?

That is a a lot of money for something that is "free" in most labs (although it is really not free). Laboratorians are not aware of IT costs.

Networking capability should be an up-front cost, rather than being an additional cost per sample (cost of establishing network connections would be the same, regardless of the number of samples processed)

Again - an outrageous price point

1. **Would you be willing to pay an extra £5 (€6.30) per sample to achieve the test result in <2hours?**

Comments:

It would be hard to identify healthcare cost savings for a 2hr versus 4hr result to justify this additional expense.

Why would you offer this service at a premium? Surely it's supposed to be patient centred care.

The clinical impact of having a results in < 2 hours, rather than < 4 hours, will depend on local organisational factors (such as patient flow, staffing, opening hours, etc)

In some cases

doesn't seem cost-effective for the sake of 2hrs

No - it either does it better than the current system or it doesn't - there should be no extra costs according to level of service

1. **Would you be willing to pay an extra £2.50 (€3.10) per sample to detect genetic indicators of resistance to trimethoprim?**

Comments:

Doubtful... A history of trimethoprim treatment is probably as useful as an indicator of resistance. Steinke et al (2001) JAC 47:781-787

Handy.

Not sure that all genetic indicators could be detected.

This type of epidemiological surveillance is useful, but not necessarily for every user of the deveice. Sentinal users may be able to provide this data

Possibly.

**OTHER COMMENTS:**

Needs to be funded so as to be cost-free clinicians using it. I know this sounds ridiculous, but the present system is under bulk contract so not pay-per-use, equipment and consumable costs would be prohibitive to an individual GP practice - we'll just have to keep waiting...!

Can you give a reference cost for the normal running of this service i a GP practice.

The ability to rapidly identify negative samples (i.e. no pathogen detected) within 1 hour would likely have a clinical impact (particularly in primary care and ED settings)
