# Supplementary material for: Criteria required for an acceptable point-of-care test for UTI detection: Obtaining consensus using the Delphi technique
Source: PLoS One. 2018 Jun 7;13(6):e0198595. doi: 10.1371/journal.pone.0198595 (PMC5991694; doi:10.1371/journal.pone.0198595)
Supplement: S4 File — (DOCX) [file pone.0198595.s006.docx]

# Supporting Information File 4: Round 2 comments from panel members.

Please note all comments are verbatim.

**Section 1: Intended use of the point-of-care test.**

**Statement 1:**

**The point-of-care test can be used within secondary care as a “one-step” test for the detection and identification of urinary pathogens when a faster result is required.**

Explanation: In comparison to a) Matrix-assisted laser desorption/ionization time-of-flight (MALDI-TOF) which identifies pathogens once detected and b) the dipstick which only detects the presence of a pathogen, this point-of-care test will detect and identify the urinary pathogens in a one-step process.

Comments:

As I understand the statement ( I don't really understand what "within secondary care" means), the test would determine if a pathogen is present but will not tell you what the pathogen is.

There are likely to be a number of factors relating to the application of a POC test in secondary care, including a potentially greater range of urinary pathogens (thus requiring a greater number of targets within the test) and interpretation of results (particularly when there is likely to be more ready access to relevant clinical expertise in secondary care, compared to primary care). Where the test may be of most use in secondary care is in identifying common urinary pathogens, to guide antimicrobial stewardship. Overall, I don't think the statement as it stands should be a key requirement for the test

This would be an ideal situation, not withstanding continued interpretation of the significance of any organism detected in urine

**Statement 2:**

**The point-of-care test will require patients’ consent (where possible and practicable) for their urine specimen as occurs with all diagnostic tests.**

Comments:

Badly worded statement and not possible to answer Could mean that proper informed consent is needed for test of could mean same level of consent needed as for any other non-invasive test. For most everyday tests infomred consent is not obtained and, in any event, level of information needed for this test is lower than (for example) blood tests. here there is only one outcome of interest but blood test might turn up any number of unexpected results that people may not wish to know I have marked neutral only because I cannot get to next page without completing

Verbal consent adequate

Implied consent

In our institution this consent is included in the "generalized consent to treat" that all patients sign. There are certain "opt out" provisions such as not having an HIV test without specific consent.

I presume "as occurs with all diagnostic tests" does not imply a requirement for written consent for the POC test

best practice

The same consent process should apply for all diagnostic tests, no additional specific consent should be necessary for different analytic modalities

**Section 2: The detection and identification of** **potential urinary pathogens.**

**Statement 3:**

**The level of detection required by the point-of-care test for the urinary pathogens is from 10^2^ to greater than 10^5^ CFU/ml as determined by the published guidelines.**

Explanation: Based on: 1. Clinical guidelines including Scottish Intercollegiate Guidelines (SIGN), European Association of Urology (EAU), and Infectious Disease Society of America (IDSA). 2. Laboratory guidelines including Cumulative techniques and procedures in clinical microbiology (Cumitech) and Public Health England. Please see table 1 for a summary of the level of detection suggested by these groups to indicate presence of urinary pathogens.

Comments:

Surely you mean the minimal level of detection....

Agree, providing test can also handle >10^5 CFU/mL rather than being limited to the specified range of 10^2 to 10^5.

100-1000 CFU is too low for a point-of-care test-it will create too much noise. More comfortable with greater than or equal to 10,000 cfu

The test should provide some indication of the detection threshold, or a semi-quantitative result

Any organisms detected in a patients urine needs to be interpreted in light of clinical presentation

**Section 3: Features and performance of the point-of-care device**

**Statement 4:**

**Only one sample will be analysed at a time, using the point-of-care test.**

Comments:

Do not want storage of samples waiting around in a clinical area for testing. Once the test is complete then the sample must be discarded.

if the equipment can analyse more than one sample at any given time and is validated to do that then i would disagree with the above statement

To avoid human error

What is the turn-around-time? If it is more than 10-15 minutes, I might not be in favor. Less than 10 minutes would be fine.

The sensitivity of the test should be sufficient to require one sample only

**Statement 5:**

**Relevant healthcare professionals can be notified of results from the point-of-care test automatically via email as an optional feature.**

Explanation: Optional because this will incur extra costs to cover integration of networking capabilities. For this to be achieved clinical governance frameworks (for example, data security and confidentiality) would need to be considered.

Comments:

only if cost effective - if recorded in patient notes - sufficient

No need for e mail extra cost and need for access to e mail on wards not practical

I think this is a good idea. Patients may also want to be automatically informed of the result using the same technology

**Section 4: Operation of the point-of-care test by user**

**Statement 6:**

**Staff operation of the point-of-care test will include:**

**-Receipt and verification of the urine sample from the patient**

Comments:

the patient may not be well enough to verify the urine sample? and staff should be able to do this

Avoid error

There needs to be a clear method for documenting that the identity of the patient has been matched to the sample

Ideally a point of care test will be carried out by a patient facing member of clinical staff such as a nurse of doctor.

**Section 5: Costs associated with the point-of-care test**

**Statement 7:**

***Would you be willing to pay £30 (€38) per sample to detect and identify the most common urinary pathogens within 4 hours (cost includes the price of the point-of-care instrument)?*** *Explanation: By comparison conventional culture methods cost around £35 (€44.45) per sample and typically take 48-120 hours for detection and identification of pathogens (cost excludes staff wages).*

Comments:

In Denmark a culture performed at the GPs office cost 6 Â£ (expenditure for GP to bye the agarplate, Flexicult)

Neutral response, given that this is likely to be a new cost in addition to routine culture. If this test replaced routine culture then my vote would be for "Strongly agree".

That is very given the cost of a urine culture is less.

For certain patient groups in certain circumstances, it would not be universally necessary to employ the point of care method

**Statement 8:**

***If the initial device cost less than £10,000 (€12,403) would you be interested in buying the device?***

Comments:

not sure as it would depend on funding available

Gp practices it would speed up diagnosis and need to attend hospitals

**Statement 9:**

***Would you be willing to pay an extra £5 (€6.30) per sample to achieve the test result in <2hours?***

Comments:

if test result required within 2h - suggests patient more unwell and decision to treat empirically may have already been made

Making it even more expensive for something that is already very expensive is not recommended.

A < 2 hour turnaround time is more likely to fit into the workflow of primary care and emergency department settings. A particularly desirable feature would be to confirm the absence of urinary pathogens within a very short time period (in which case, confirming a positive within < 4 hours may be acceptable)

**Statement 10:**

***Would you be willing to pay an extra £2.50 (€3.10) per sample to detect genetic indicators of resistance to trimethoprim which may better inform appropriate antibiotic treatment?***

Comments:

We don't use trimethoprim empirically any more due to prevalence of resistance.

Not sure what the current practice is in Europe but my guess fluorquinolones are much more widely used in US and detection of resistance to them seems more important.

This feature would be particularly useful if the result is available within a timeframe that fits with primary care and emergency department workflow
